# Exosomes and Their Therapeutic Potentials of Stem Cells

**DOI:** 10.1155/2016/7653489

**Published:** 2015-12-06

**Authors:** Chao Han, Xuan Sun, Ling Liu, Haiyang Jiang, Yan Shen, Xiaoyun Xu, Jie Li, Guoxin Zhang, Jinsha Huang, Zhicheng Lin, Nian Xiong, Tao Wang

**Affiliations:** ^1^Department of Neurology, Union Hospital, Tongji Medical College, Huazhong University of Science and Technology, Wuhan, Hubei 430022, China; ^2^Department of Interventional Neuroradiology, China National Clinical Research Center for Neurological Diseases, Beijing Tiantan Hospital, Capital Medical University, Beijing 100050, China; ^3^Department of Psychiatry, Harvard Medical School, Division of Alcohol and Drug Abuse, and Mailman Neuroscience Research Center, McLean Hospital, Belmont, MA 02478, USA

## Abstract

Exosomes, a group of vesicles originating from the multivesicular bodies (MVBs), are released into the extracellular space when MVBs fuse with the plasma membrane. Numerous studies indicate that exosomes play important roles in cell-to-cell communication, and exosomes from specific cell types and conditions display multiple functions such as exerting positive effects on regeneration in many tissues. It is widely accepted that the therapeutic potential of stem cells may be mediated largely by the paracrine factors, so harnessing the paracrine effects of stem and progenitor cells without affecting these living, replicating, and potentially pluripotent cell populations is an advantage in terms of safety and complexity. Ascending evidence indicated that exosomes might be the main components of paracrine factors; thus, understanding the role of exosomes in each subtype of stem cells is far-reaching. In this review, we discuss the functions of exosomes from different types of stem cells and emphasize the therapeutic potentials of exosomes, providing an alternative way of developing strategies to cure diseases.

## 1. Introduction

Regenerative medicine aims to improve the regeneration of damaged, malfunctioning, and missing tissue and organs [1]. Mounting evidence supports that stem cell therapies may be promising in this field on the basis of potential therapeutic use of stem cells in damaged organs such as the myocardium after heart infarction, stroke, spinal cord injury, retina diseases, and damaged liver [2–4]. In addition, stem cells-based therapy may be a prospective way for diseases that are irreversible and incurable at present [5]. Specifically, regenerative medicine contains two goals: one is efficiently and safely transferring stem cells into injured organs and tissues, which may replace the transplantation of the entire organ in the near future; the other is to develop strategies in order to improve the regenerative potential and function of adult stem cells residing in various organs [6]. In the last decades, numerous preclinical studies confirmed the therapeutic potentials of stem cells. Stem cells involving embryonic stem cells (ESCs), induced pluripotent stem cells (iPSCs), and adult stem cells manifest respective merits and drawbacks. Some types of stem cells are being evaluated in clinical trials with promising results [7]. These stem cells such as mesenchymal stem cells (MSCs) are relatively safe, but therapeutic strategies avoiding direct use of living stem cells are more likely to provide a safer way to prevent disease progression. Although direct and indirect mechanisms such as growth factors and cytokines have accounted for the therapeutic effects, paracrine secretion seems to play a predominant role. A key component of paracrine secretion is extracellular vesicles (EVs), particularly the exosome fraction that mainly contributes to the action of stem cells in which genetic information can be horizontally transferred between stem cells and tissue-injured cells. On the basis of the ability of microvesicles (MVs) to mimic stem cell properties, it is speculated that stem cell-derived MVs especially exosomes represent a relevant therapeutic option in regenerative medicine. In this review, we summarize the roles that MVs especially exosomes play in each type of stem cells.

## 2. Characteristics of Exosomes and Function

**Figure 1:**
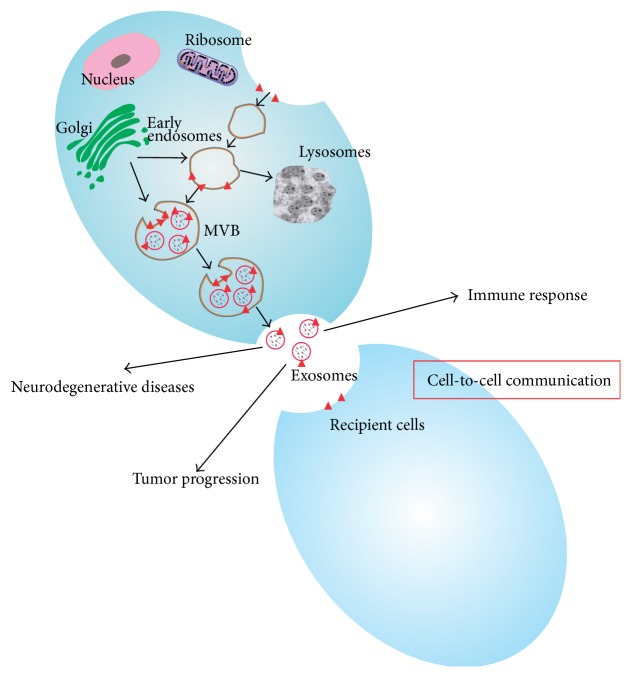
Biogenesis and action of exosomes. Exosomes are formed by inward budding of membrane of the multivesicular bodies (MVBs); when MVBs fused with the membranes, the exosomes are released. Exosomes can deliver lipids, proteins, and nucleic acid to recipient cells when circulating in the extracellular space. Exosomes are important mediators of intercellular communication and play significant roles in immune response, tumor progression, and neurodegenerative disease among others.

**Figure 2:**
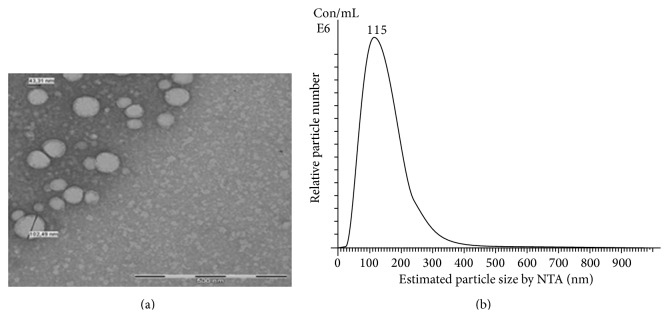
Characteristics of exosomes isolated from the MSCs [27]. (a) Representing exosomes identified by the transmission electron microscopy (TEM); the size ranges from 40 to 120 nm, scale bar 500 nm. (b) Showing the distribution of diameters of exosomes measured by the Nanosight (NTA). The most common diameter was around 115 nm.

Exosomes are one of the several groups of EVs which include ectosomes secreted directly from the plasma membranes and apoptotic bodies released from dying cells. Exosomes originate from the inward budding of the cell membranes followed by formation of multivesicular bodies (MVBs). When MVBs fuse with the plasma membranes, exosomes are released (Figure 1). Since they were discovered to be released from sheep reticulocytes, exosomes were once defined as unwanted proteins secreted from the cells and manifested as a membrane vesicle [8]. Currently, exosomes have been verified to be secreted from various cells including B cells [9], T cells [10], dendritic cells [11], platelets [12], the Schwann cells [13], tumor cells [14], cardiomyocytes [15], endothelial cells [16], and stem cells [17] among others. Moreover, exosomes are found in physiological fluids such as urine [18], plasma [19], and cerebral fluid [20] and even in organs such as thymus [21]. Exosomes are characterized by their diameters ranging from 30 to 120 nm and with a density in sucrose of 1.13–1.19 g/mL. Their membranes contain abundant cholesterol, sphingomyelin, ceramide, and lipid rafts. Besides, exosomes are enriched with various nucleic acids including mRNAs, microRNAs (miRNAs), and other noncoding RNAs [22]. These RNAs can be taken up by neighboring cells or remote cells, subsequently modulating recipient cells; on the other hand, RNAs are protected from degradation after being packed into the exosomes or microvesicles, which altogether results in increased attention to exosomes and the carried RNAs. On this basis, an increasing number of mRNAs and miRNAs have been discovered in different cell-derived exosomes. Most exosomes have conserved a set of proteins such as heat shock proteins, HSP70 [23] and HSP90 [24], certain members of the tetraspanin superfamily of proteins, especially CD9, CD63, CD81, and CD82 [25], multivesicle related proteins such as Alix and TSG-101, and membrane transportation and merging proteins such as Rab GTPase and flotillin. In addition, exosomes contain unique tissue proteins that may reflect their cellular source. Mathivanan and Simpson [26] set up the ExoCarta, a freely accessible database listing proteins and RNAs that have been found in exosomes. The representative characteristics of exosomes isolated from MSCs by transmission electron microscopy (TEM) and nanoparticle tracking analysis (NTA) are shown in Figure 2 [27]. When referring to the function of exosomes (Table 1), though not clarified yet, most of the time we correlate it with intercellular communication vehicle for modulating cellular processes [9, 11, 25, 28, 29]. For instance, exosomes participate in noninfectious diseases such as cancer [30] and degenerative diseases like Parkinson's disease and Alzheimer's disease [31, 32] as well as the process of immune response [33] (Figure 1).

## 3. Exosomes and Their Roles in Stem Cells

Stem cells are kind of cells with self-renewal and multiplex differentiation potential and can be differentiated into various kinds of adult cells, like the ESCs, iPSCs, and somatic stem cells [34, 35]. Previously, cell therapy attracts much attention but encountered many realistic problems such as the possibility of immune rejection and ethical issues though pluripotent ESCs have long been deemed as an ideal choice for regenerative medicine. As a result of the ability of circumventing the problems associated with ESCs, iPSCs have gained increasing attention. However, despite their promising potential, there are still many hurdles that should be overcome before iPSCs-based therapies are put into clinical practice [36]. Specifically, therapeutic application of iPSCs may involve the risk of teratoma formation and may cause genetic modification, which could possibly give rise to various obstacles [37, 38]. Accordingly, a better stem cell type is needed urgently. MSCs, a type of adult stem cells appearing safe, have been widely used in a variety of clinical experiments [39]. One of the problems is the limited number of transplanted MSCs in animal models such as models of kidney injury [40], lung injury [41], and acute myocardial infarction [42] after administration and rare differentiation into appropriate cell types [43–45]; besides, the therapeutic effect of MSCs may not correlate with the engraftment, differentiation, and cell fusion when stem cells are added to the target cells [46, 47]. These phenomena may indicate that the MSCs exert their therapeutic effects through the effects of secreted factors. Exosomes, an important part of active components of the factors, are paid increasing attention and studies have shown that the exosomes derived from stem cells imitate the phenotype of parent stem cells, holding a therapeutic potential for various diseases [48–51]. Stem cells like the iPSCs, ESCs, hemopoietic stem cells (HSCs), mesenchymal stem cells (MSCs), and neural stem cells (NSCs) were all capable of secreting exosomes [52, 53]. In addition, the exchange between the exosomes and target cells are bidirectional; on the one hand, damaged cells secrete exosomes containing cell-specific miRNAs and, after internalization within the stem cells, stem cells begin to differentiate and acquire tissue specific cell types; on the other hand, MVs released from stem cells may confer a stem-cell-like phenotype to injured cells, consequently activating self-regenerative programs [54]. In the following part, the exosomes and the potential use in different types of stem cells including the ESCs and adult stem cells are discussed.

## 4. ESCs

ESCs are pluripotent, self-renewing cells derived from the inner cell mass of developing blastocyst [55]. ESCs have been shown to represent an abundant source of MVs containing critical components supporting self-renewal and expansion of stem cells [56, 57]. In addition, they contain cellular signaling molecules such as different kinds of mRNAs, microRNAs, and proteins. Ratajczak et al. [57] proved that exosomes secreted from the ESCs enhanced survival and improved expansion of murine hematopoietic progenitor cells (HSPCs) and enhanced expression of early pluripotent genes by transferring mRNAs and proteins. Yuan et al. [58] characterized the RNAs and protein contents of MVs and indicated that exosomes could be engineered to carry exogenously expressed mRNAs and proteins such as green fluorescent protein (GFP). Moreover, exosomes could alter the expression of genes by transferring microRNAs and GFP contained in MVs when docking and fusing with other ESCs. Consequently, ESCs-derived MVs especially exosomes might be useful therapeutic tools for transferring mRNAs, microRNAs, proteins, and siRNAs to cells and important mediators of signaling within stem cell niches. Additionally, MVs released from the ESCs could induce differentiation and pluripotency in their target Muller cells [59], thus initiating an early retinogenic process of differentiation. Jeong et al. [60] showed that MVs engineered from ESCs could enhance cell proliferation and potentially contribute to recovery or wound healing process of tissues. Simultaneously, we should be prudent toward the clinical use of MVs derived from the ESCs, which was best exemplified by Kubikova et al.'s study [61] in which they had a proteomic profiling of MVs derived from human ESCs. This study confirmed the role of MVs in communicating between human ESCs. More importantly, they highlighted a potential risk toward the clinical application of human ESCs on the basis of their finding that immunogenic membrane domains and infectious particles were carried by the MVs as well.

## 5. Adult Stem Cells

### 5.1. MSCs

MSCs are ubiquitously expressed in not only many tissues of mesodermal origin such as bone marrow, adipose, muscle, or bone but also many tissues isolated from brain, liver, spleen, kidney, lung, thymus, and pancreas [98]. MSCs harbor the potential to differentiate into stromal support cells and secrete factors to support the stroma or other cells [99, 100]. The easy procedures for their expansion* in vitro* and their presence in numerous tissues make MSCs the most studied adult stem cells in regenerative medicine. The therapeutic potential and safety of MSCs have been extensively studied and numerous clinical trials are published with additional ones under trail. Moreover, the therapeutic potential of MSCs may be attributable to the paracrine factors contained in MVs. MSCs are the most prolific producer compared with other cell types known to produce exosomes [101]. Extensive studies demonstrated that MVs especially exosomes derived from MSCs could repair injured tissues. For example, the MSCs were shown to secrete exosomes that were cardioprotective and preserved cardiac performance in AMI models [17, 62]. Moreover, human MSCs-derived MVs were therapeutically effective in animal models of acute lung injury induced by endotoxin [63]. MSCs exosomes showed protective effects in acute kidney injury model as well [64]. In addition, they could facilitate cutaneous wound healing by promoting collagen synthesis and angiogenesis [69]. After rats were subjected to traumatic brain injury, MSCs exosomes could promote functional recovery and neurovascular remodeling [70] and, in neurological diseases such as stroke, MSCs exosomes also showed their therapeutic potential, as depicted in Xin et al.'s [71] study in which systemic administration with MSCs exosomes promoted functional recovery, neurite remodeling, neurogenesis, and angiogenesis in animal models of stroke.

MSCs are able to secrete immunologically active exosomes [72] and thus exert immunomodulatory effects on the differentiation, activation, and function of different lymphocyte subsets [73], suggesting that MSCs-derived exosomes can be considered as a way of treating inflammation-related diseases. Since MSCs are increasingly being used for the treatment of acute and chronic graft versus host disease (GVHD) via their immunomodulatory effects, the clinical value is fantastic when we make the most of the therapeutic potential of MSCs-derived exosomes if exosomes are involved in the immunomodulatory mechanisms. Besides, the MSCs exosomes may also be an ideal candidate for allogenetic cell-based therapy, as a result of the low immunogenicity in MSCs [74].

Another therapeutic potential of MSCs-derived MVs that is prospective is their roles in delivering drugs [75]. A recent study demonstrated that MSCs could package and deliver active drugs through their MVs [76], paving the way of using MSCs to develop new drugs with increased efficacy and homing capacity.

It is known that cell-cell communication plays an important role in the action of MVs-derived from the MSCs, especially the exosomes. The MVs can modify the target cells through surface receptor interactions and transfer of inner proteins, mRNAs, and miRNAs. Overexpression of GATA4 could potentiate the cardioprotection of MSCs, using cell-free methods. Yu et al. [102] highlighted the importance of MSCs overexpressing GATA-4-derived exosomes in cardiac protection and the exosomes released antiapoptotic miRNAs that regulated various cell survival signaling pathways. When the MSCs were subjected to an ischemic condition, the exosomes were enriched with miR-22 and could transfer this miRNA to cardiomyocytes, eventually improving cardiac function after myocardial infarction [103]. Besides, in animal models of stroke, it was the miR-133b transferred to astrocytes and neurons mediated by the MSCs exosomes that benefited neurite remodeling and functional recovery [104]. Other exosomes like these derived from the gastric cancer tissue-derived MSCs delivered miR-221 to HGC-27 cells, promoting gastric cancer proliferation and migration [105]. Currently, methods are available to characterize the components of MVs derived from the MSCs. Eirin et al. [106] characterized the RNA cargo of MVs derived from the porcine adipose-tissue MSCs and indicated that MVs were selectively enriched with different kinds of RNAs. These studies suggested that MVs conveyed gene regulatory information to modulate angiogenesis, adipogenesis, and other cell pathways in recipient cells, which altogether provide the theoretical basis of altering the content of MSCs exosomes.

Moreover, exosomes act as an important mediator of cell-to-cell communication also in the tumor microenvironment. Exosomes derived from the cancer cells could affect the differentiation of MSCs in which MSCs were more likely to change into carcinoma-associated fibroblasts [70, 107, 108]. For instance, it was shown that exosomes derived from human bone MSCs could promote tumor growth* in vivo* [65]. Additionally, human MSCs supported breast cancer cell proliferation and promoted migration via the exosomes transporting tumor regulatory microRNAs, proteins, and metabolites and might affect the signaling pathway [66, 67], similar to the roles of exosomes derived from bone marrow MSCs towards the multiple myeloma progression [68].

Other studies paid attention to the antitumor effect of MSCs exosomes. Specifically, Lee et al. [77] demonstrated that MSC-derived exosomes could inhibit tumor growth and suppress angiogenesis by downregulating vascular endothelial growth factor (VEGF) mediated by the exosome-delivered miR-16. By modifying the content of exosomes such as overexpressing miR-146, Katakowski et al. [78] indicated that exosomes significantly inhibited the growth of brain tumor. It was once reported that bone MSCs possessed potential antitumor activity but the action was insufficient and weak [109]; Ma et al. [79] generated the new method of combining bone MSCs with the tumor-derived exosomes which was later confirmed to enhance MSCs' antitumor activity. This exploration provides a promising method regarding antitumor activity that needs further examination.

### 5.2. Neural Stem Cells and Neural Progenitor Cells

Neural stem cells (NSCs) are undifferentiated cells with the potential to self-renew and give rise to all the main cell types of central nervous system including the neurons, astrocytes, and oligodendrocytes while neural progenitor cells (NPCs) have less differentiation potential and limited renewal capacity [110, 111]. Owing to their characteristics, NSCs/NPCs are selected as tools to study the mechanism of disease conditions regarding the central nervous system. For instance, abnormal differentiation of NPCs contributed to the pathophysiology of fragile X syndrome [112]. Coordinated signals contributed to the origin or amplification of neuropathological development of NSCs, but the regulatory mechanism remained elusive. Feliciano et al. [81] pointed out that embryonic CSF nanovesicles especially exosomes contained proteins and microRNAs that host key determinants in the insulin-like growth factor pathway which regulated NSC proliferation [80, 82]. Lee et al. [113] demonstrated that MSCs promoted neural cells' differentiation by delivering exogenous microRNAs to human NPCs, providing a theoretical basis of the potential by efficient delivery of microRNAs into the brain. On the other hand, the NSCs were able to secrete large amounts of exosomes [114]. One study showed that exosomes facilitated the process in which the virus enters the cells [83] and this process could be hampered by antibody targeting molecules expressed on the exosomes [84]. These data implied an alternative way regarding the virus/exosome path, which might help develop therapeutics to reduce the viral infection. In order to characterize the exosomes in human NSCs, Kang et al. [85] adopted the method of flow field-flow fractionation and nanoflow liquid chromatography-tandem mass spectrometry, identifying 103 proteins in the exosomes, among which the diameter larger than 50 nm was morphologically distinct from those smaller than 50 nm and the protein contents of each type were different. Importantly, the results showed that the exosomes contained polymyositis/scleroderma autoantigen 2 (PM/Scl2) which was specific to systemic sclerosis (scleroderma), indicating that exosomes might participate in triggering autoimmunity. We speculate that the exosomes derived from the NPCs might be applied to numerous neurological diseases in the near future.

### 5.3. EPCs

Endothelial progenitor cells (EPCs) are stem cells with the capacity to differentiate into endothelial cells [115], which forms the inner lining of a blood vessel. The EPCs exosomes could steer angiogenesis in which exosomes derived from EPCs bind to *α*4 and *β*1 integrins expressed on the MV surface, promoting endothelial cell survival, proliferation, and organization both* in vitro* and* in vivo* [86]. Furthermore, this process was closely related to mRNAs transfer because MVs pretreatment with RNase abrogated the angiogenic activity. Cantaluppi et al. [87] found that the exosomes released from the EPCs protected human islets by enhancing their vascularization and it was the microRNAs shuttled by the exosomes that contributed to their angiogenic effects. In addition, the data indicated that exosomes released from EPCs protected acute kidney injury in rat models of ischemia-reperfusion injury via transferring miRNAs. Specifically, exosomes derived from the EPCs contained abundant miR-126 and miR-296 which promoted the angiogenesis and antiapoptosis [116]. Later research confirmed the angiogenesis of exosomes derived from EPCs in murine model of hindlimb ischemia [88]. All abovementioned studies indicated that the contents of exosomes determined the action and it was reconfirmed, in other disease models like hypoxia/reoxygenation induced human brain microvascular endothelial cell injury, that the exosomes yielded two distinct effects via two different kinds of carried RNAs associated with ROS production and PI3K/eNOS/NO pathway contained in the exosomes [89]. Additionally, exosomes derived from EPCs exerted protective effects on cardiomyocytes against angiotensin II- (Ang II-) induced hypertrophy and apoptosis [90]. Therefore, exosomes derived from EPCs can be a promising therapeutic agent.

### 5.4. HSCs and HSPCs

HSCs are stem cells with the function of producing all lineages of blood cells and own the capacity of self-renewal [117] whereas abnormal differentiation may lead to chronic myeloid leukemia (CML). HSCs-secreted exosomes contain the stem cell marker prominin-1 (CD133) which played important roles in maintaining stem cell properties and hosting key determinants in the endocytic-exocytic pathway [91]. CD133+ cells purified from hematopoietic tissues are another potential source of stem cells; MVs derived from these cells were proved to express mRNAs of several antiapoptotic and proangiopoietic factors which promoted angiogenesis, providing a theoretical basis for application of purified CD133+ cells in regenerative medicine [92]. Salvucci et al. [93] indicated that exosomes from G-CSF (granulocyte colony-stimulating factor) mobilized bone marrows contained abundant miR-126 and G-CSF, promoting the accumulation of exosomes in the bone marrow. Moreover, miR-126 delivered by exosomes reduced the expression of vascular cell adhesion molecule-1 (VCAM1) which was crucial to the retention of hematopoietic progenitor cells (HSPCs) in the bone marrow. Then, the reduced level of VCAM1 led to the mobilization of hematopoietic stem/progenitor cells (HSCs/HSPCs) from the bone marrow to the peripheral blood. In addition, CML-derived exosomes promoted the proliferation and survival of tumor cells via an autocrine action in antiapoptotic pathways and this process was mediated by selectively expressed miRNAs as well [94, 118]. When referring to therapeutic effect, Ratajczak et al. [92] found that HSC/HSPCs-secreted exosomes expressed mRNAs of several antiapoptotic and proangiopoietic factors like the VEGF, insulin growth factor-1, basic fibroblast growth factor, and interleukin-8. These mRNAs exert antiapoptotic property, increase the survival of endothelial cells, and stimulate their proliferation and tube formation. Since the CML could secrete exosomes, leukemia cell-derived exosomes-based vaccines might be a promising strategy for enhancing survival in patients suffering from chemotherapy and HSCs transplantation [94]. Accordingly, we postulate that improvements will be seen in terms of therapeutic effects toward the blood diseases such as the CML in the near future.

### 5.5. CPCs and Other Stem Cell Types

Cardiac progenitor cells (CPCs) are another attractive candidate for treatment of myocardial diseases. The process of CPCs-mediated cardioprotection can be attributed to both cardiovascular lineage differentiation and paracrine effects [119–121]. The exosomes are the key components of the paracrine factors in both human [122] and mouse CPCs [95] and exert cardiac protection involving microRNAs both* in vitro* and* in vivo*. Gray et al. [96] demonstrated that CPCs secreted proregenerative exosomes in response to hypoxia, and 11 miRNAs were upregulated compared to normal exosomes. Moreover, exosomes derived from the hypoxic CPCs improved cardiac function and reduced fibrosis. Ong et al. [97] showed that CPCs-overexpressing hypoxia-inducible factor-1 (HIF-1) improved the survival of transplanted CPCs and these results were attributed to the high levels of miR-126 and miR-120 contained in the exosomes that activated prosurvival kinases and induced a glycolytic switch in recipient CPCs. These data indicated that transferring of microRNAs from host cells to transplanted cells might represent a promising way to improve the survival of transplanted cells. Vrijsen et al. [15] demonstrated that CPCs released exosomes into their environment, stimulating migration of endothelial cells in an* in vitro* scratch wound assay, and the mechanism was related to extracellular matrix metalloproteinase inducer mediated activation.

Other stem cells-derived exosomes such as those derived from human liver stem cells (HLSCs) contributed to self-renewal and expansion of stem cells [57]; besides, exosomes derived from HLSCs activated a proliferative program in remnant hepatocytes after hepatectomy by horizontal transferring of specific mRNAs, eventually accelerating liver regeneration* in vivo* [123]. In addition, human CD34(+) stem cells secreted exosomes that displayed an independent angiogenic activity both* in vitro* and* in vivo* [124]. All these results demonstrate that exosomes from stem cells might represent a significant component of the paracrine effect of progenitor cell transplantation for therapeutic angiogenesis.

## 6. Conclusion

In summary, exosomes can be released by various kinds of stem cells and are able to modify the function of the receptor cells and tissues. Compared with stem cells, which may cause abnormal differentiation and tumor formation, the exosomes mediated therapy harbors a more promising future. Some diseases including idiopathic pulmonary fibrosis are currently incurable, but MVs especially the exosomes have shown therapeutic potentials [125]. However, there are still challenges to overcome in studies of exosomes. The most common method in isolation of exosomes is still ultracentrifugation which is time-consuming and requires a large amount of cells and biological fluid. Although commercial exosome extraction reagents are now available and yield high numbers of exosomes, the products still need purification as they contain non-EV contaminants such as lipoproteins [126]. There are other open areas such as the process where the exosomes choose respective cargo to transport, the way the cells take up the exosomes, and how many types of exosomes warrant further investigations. When referring to the exosomes released by the stem cells, the definite mechanisms of the action and specific therapeutic potential of each subtype still need further efforts.

## Figures and Tables

**Table 1:** The function of exosomes derived from different types of stem cells.

Type	Physiological function	Therapeutic function	Pathological function	References
ESCs	Self-renewal and expansion	Enhancing survival and expansion of HPSCs		[56–58]
		Inducing gene expression changes in Muller cells of the retina		[59]
		Wound recovery		[60]

MSCs	Promoting MSCs proliferation	Repairing injured issues like heart, lung, and kidney	Promoting tumor growth	[17, 62–68]
		Cutaneous wound healing, TBI, and stroke		[69–71]
		Exerting immunomodulatory role on lymphocyte subsets		[72, 73]
		Allogenetic cell-based therapy		[74]
		Delivering drugs		[75, 76]
		Antitumor		[77–79]
		Promoting NPCs differentiation		[80]

NSCs	Regulating NSCs proliferation	Antibody targeting exosomes that may reduce viral infection	Neuropathological development of NSCs	[81–84]
			Triggering autoimmunity	[85]

EPCs	Promoting endothelial survival	Protecting human islets		[86, 87]
		Steering angiogenesis in acute kidney injury		[87]
		Promoting angiogenesis in hindlimb ischemia		[88]
		Protecting H/R induced endothelial cell injury		[89]
		Protecting cardiomyocytes		[90]

HSCs	Maintaining stem cell property	Increasing survival of endothelial cells		[91–93]
		MVs of CD133+ cells derived from hematopoietic tissues promote angiogenesis		[92]
		Vaccines for LEX that may enhance survival of patients with leukemia		[94]

CPCs	Self-renewal and differentiation	Cardioprotection promoting migration of endothelial cells		[95–97] [15]

Note: ESCs: embryonic stem cells; HPSCs: hematopoietic progenitor cells; MSCs: mesenchymal stem cells; TBI: traumatic brain injury; NPCs: neural progenitor cells; NSCs: neural stem cells; EPCs: endothelial progenitor cells; H/R: hypoxia/reoxygenation; HSCs: hematopoietic stem cells; MVs: microvesicles; LEX: leukemia cell-derived exosomes; CPCs: cardiac progenitor cells.

## Abbreviations

MVBs: Multivesicular bodies
ESCs: Embryonic stem cells
iPSCs: Induced pluripotent stem cells
MSCs: Mesenchymal stem cells
EVs: Extracellular vesicles
MVs: Microvesicles
TEM: Transmission electron microscopy
NTA: Nanoparticle tracking analysis
HSCs: Hemopoietic stem cells
NSCs: Neural stem cells
HSPCs: Hematopoietic progenitor cells
G-CSF: Granulocyte colony-stimulating factor
VCAM1: Vascular cell adhesion molecule-1
GFP: Green fluorescent protein
TBI: Traumatic brain injury
GVHD: Graft versus host disease
VEGF: Vascular endothelial growth factor
NPCs: Neural progenitor cells
PM/Scl2: Polymyositis/scleroderma autoantigen 2
EPCs: Endothelial progenitor cells
CML: Chronic myeloid leukemia
HSPCs: Hematopoietic progenitor cells
CPCs: Cardiac progenitor cells
HIF-1: Hypoxia-inducible factor-1
HLSCs: Human liver stem cells.
